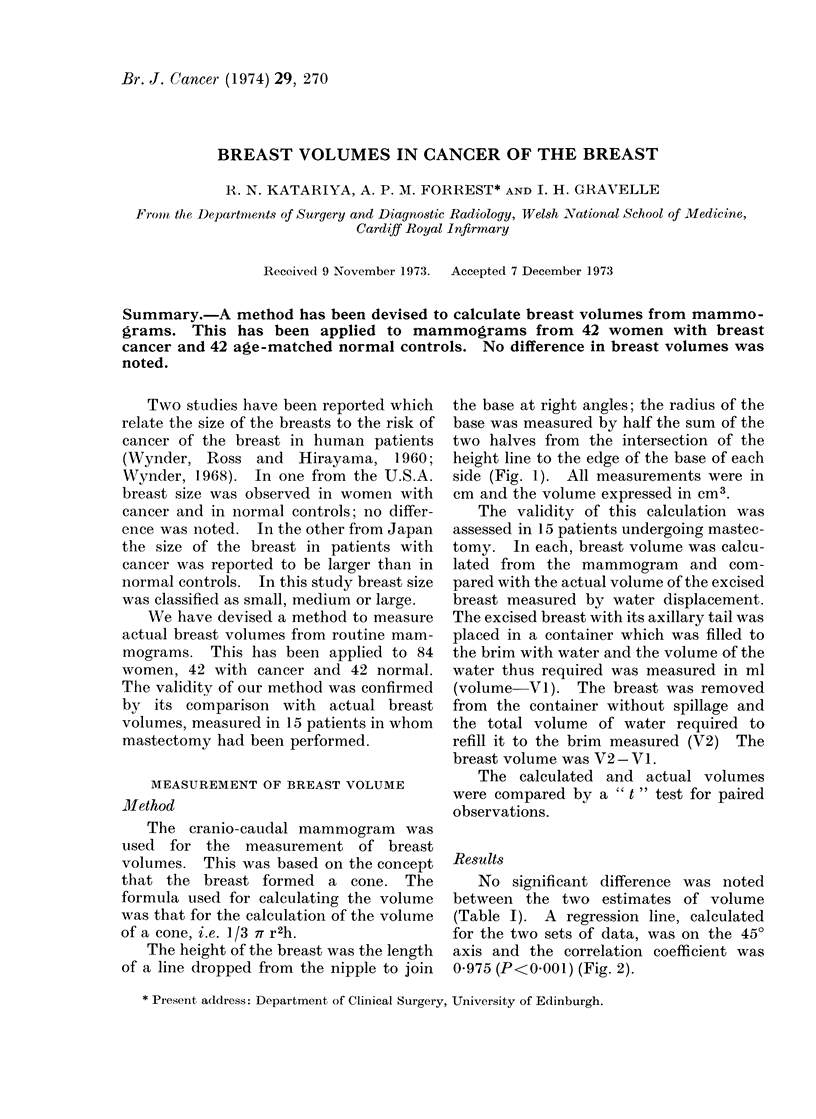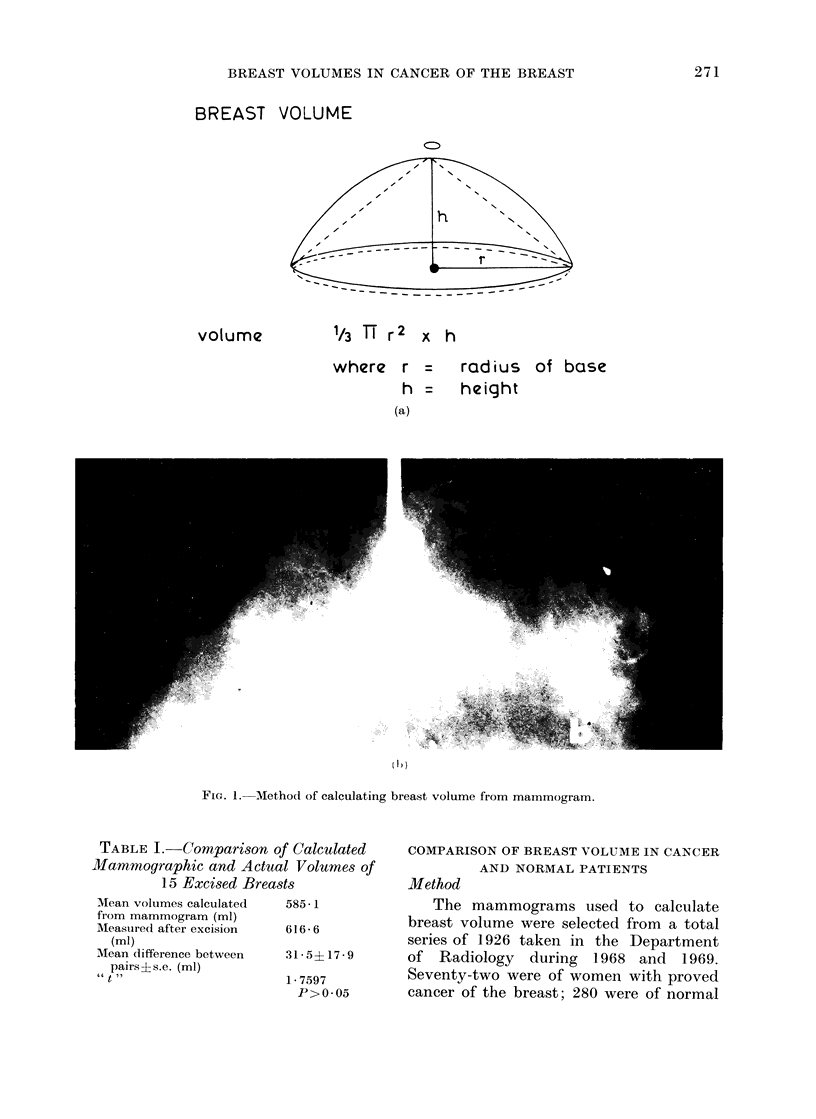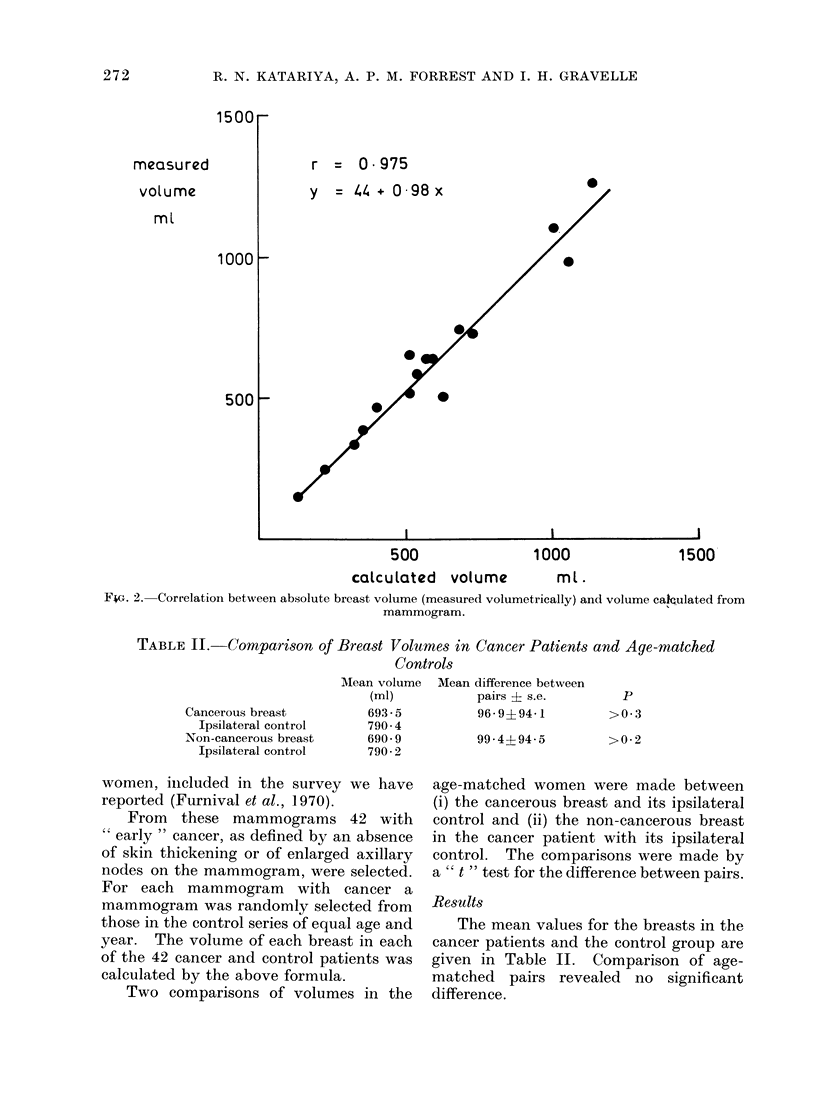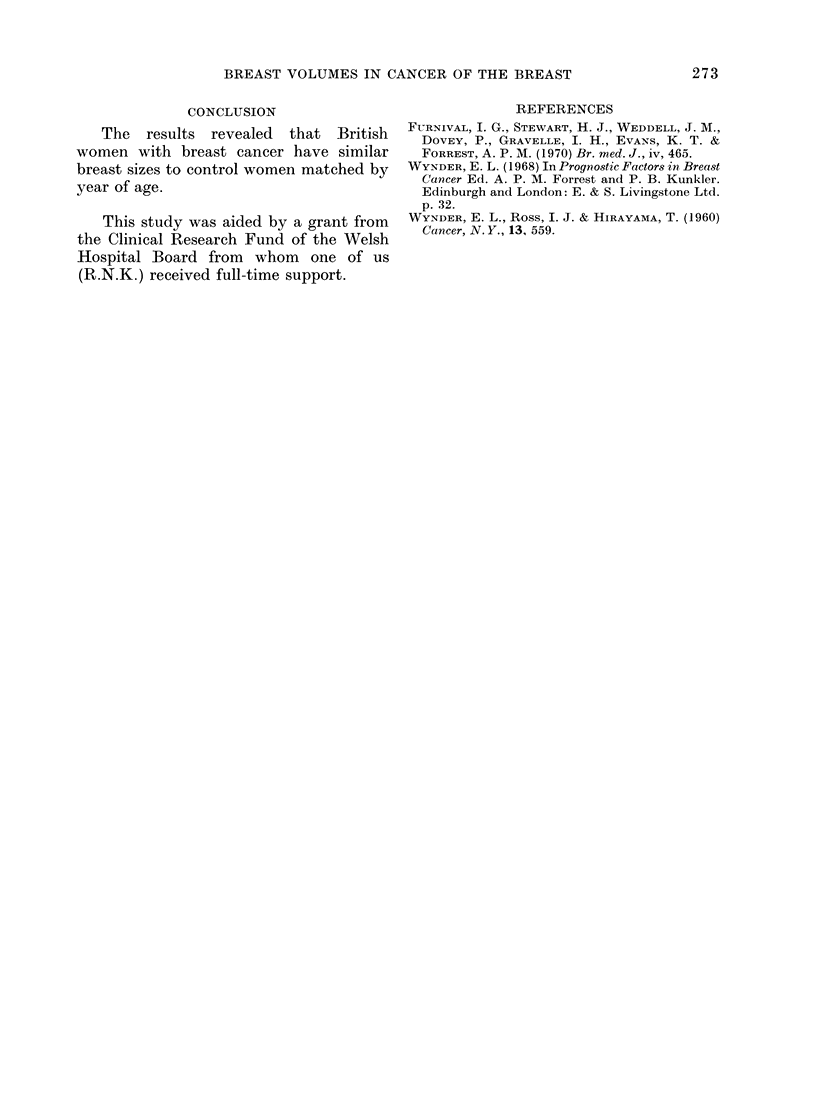# Breast Volumes in Cancer of the Breast

**DOI:** 10.1038/bjc.1974.66

**Published:** 1974-03

**Authors:** R. N. Katariya, A. P. M. Forrest, I. H. Gravelle

## Abstract

**Images:**


*Br. J. Cancer* (1974) **29**, 270

### BREAST VOLUMES IN CANCER OF THE BREAST

R. N. KATARIYA, A. P. M. FORREST* AND I. H. GRAVELLE

*From the Departments of Surgery and Diagnostic Radiology, Welsh National School of Medicine, Cardiff Royal Infirmary*

Received 9 November 1973. Accepted 7 December 1973

**Summary.—A method has been devised to calculate breast volumes from mammograms. This has been applied to mammograms from 42 women with breast cancer and 42 age-matched normal controls. No difference in breast volumes was noted.**

Two studies have been reported which relate the size of the breasts to the risk of cancer of the breast in human patients (Wynder, Ross and Hirayama, 1960; Wynder, 1968). In one from the U.S.A. breast size was observed in women with cancer and in normal controls; no difference was noted. In the other from Japan the size of the breast in patients with cancer was reported to be larger than in normal controls. In this study breast size was classified as small, medium or large.

We have devised a method to measure actual breast volumes from routine mammograms. This has been applied to 84 women, 42 with cancer and 42 normal. The validity of our method was confirmed by its comparison with actual breast volumes, measured in 15 patients in whom mastectomy had been performed.

### MEASUREMENT OF BREAST VOLUME

### *Method*

The cranio-caudal mammogram was used for the measurement of breast volumes. This was based on the concept that the breast formed a cone. The formula used for calculating the volume was that for the calculation of the volume of a cone, *i.e.* $1/3\ \pi\ r^2h$.

The height of the breast was the length of a line dropped from the nipple to join the base at right angles; the radius of the base was measured by half the sum of the two halves from the intersection of the height line to the edge of the base of each side (Fig. 1). All measurements were in cm and the volume expressed in $cm^3$.

The validity of this calculation was assessed in 15 patients undergoing mastectomy. In each, breast volume was calculated from the mammogram and compared with the actual volume of the excised breast measured by water displacement. The excised breast with its axillary tail was placed in a container which was filled to the brim with water and the volume of the water thus required was measured in ml (volume—V1). The breast was removed from the container without spillage and the total volume of water required to refill it to the brim measured (V2) The breast volume was V2 − V1.

The calculated and actual volumes were compared by a " *t* " test for paired observations.

### *Results*

No significant difference was noted between the two estimates of volume (Table I). A regression line, calculated for the two sets of data, was on the 45° axis and the correlation coefficient was 0·975 ($P<0{\cdot}001$) (Fig. 2).

* Present address: Department of Clinical Surgery, University of Edinburgh.

FIG. 1.—Method of calculating breast volume from mammogram.

TABLE I.—*Comparison of Calculated Mammographic and Actual Volumes of 15 Excised Breasts*

| | |
|---|---|
| Mean volumes calculated from mammogram (ml) | 585·1 |
| Measured after excision (ml) | 616·6 |
| Mean difference between pairs ± s.e. (ml) | 31·5 ± 17·9 |
| " $t$ " | 1·7597<br>$P > 0·05$ |

COMPARISON OF BREAST VOLUME IN CANCER AND NORMAL PATIENTS

*Method*

The mammograms used to calculate breast volume were selected from a total series of 1926 taken in the Department of Radiology during 1968 and 1969. Seventy-two were of women with proved cancer of the breast; 280 were of normal

FIG. 2.—Correlation between absolute breast volume (measured volumetrically) and volume calculated from mammogram.

TABLE II.—*Comparison of Breast Volumes in Cancer Patients and Age-matched Controls*

| | Mean volume (ml) | Mean difference between pairs ± s.e. | *P* |
|---|---|---|---|
| Cancerous breast | 693·5 | 96·9±94·1 | >0·3 |
| Ipsilateral control | 790·4 | | |
| Non-cancerous breast | 690·9 | 99·4±94·5 | >0·2 |
| Ipsilateral control | 790·2 | | |

women, included in the survey we have reported (Furnival *et al.*, 1970).

From these mammograms 42 with " early " cancer, as defined by an absence of skin thickening or of enlarged axillary nodes on the mammogram, were selected. For each mammogram with cancer a mammogram was randomly selected from those in the control series of equal age and year. The volume of each breast in each of the 42 cancer and control patients was calculated by the above formula.

Two comparisons of volumes in the age-matched women were made between (i) the cancerous breast and its ipsilateral control and (ii) the non-cancerous breast in the cancer patient with its ipsilateral control. The comparisons were made by a " *t* " test for the difference between pairs.

*Results*

The mean values for the breasts in the cancer patients and the control group are given in Table II. Comparison of age-matched pairs revealed no significant difference.

### CONCLUSION

The results revealed that British women with breast cancer have similar breast sizes to control women matched by year of age.

This study was aided by a grant from the Clinical Research Fund of the Welsh Hospital Board from whom one of us (R.N.K.) received full-time support.

### REFERENCES

FURNIVAL, I. G., STEWART, H. J., WEDDELL, J. M., DOVEY, P., GRAVELLE, I. H., EVANS, K. T. & FORREST, A. P. M. (1970) *Br. med. J.*, iv, 465.

WYNDER, E. L. (1968) In *Prognostic Factors in Breast Cancer* Ed. A. P. M. Forrest and P. B. Kunkler. Edinburgh and London: E. & S. Livingstone Ltd. p. 32.

WYNDER, E. L., ROSS, I. J. & HIRAYAMA, T. (1960) *Cancer, N.Y.*, **13**, 559.